# Governance of health research in four eastern and southern African countries

**DOI:** 10.1186/s12961-021-00781-3

**Published:** 2021-10-13

**Authors:** Pamela A. Juma, Catherine M. Jones, Rhona Mijumbi-Deve, Clare Wenham, Tiny Masupe, Joelle Sobngwi-Tambekou, Godfrey Biemba, Namuunda Mtombo, Justin Parkhurst

**Affiliations:** 1 Nairobi, Kenya; 2 Department of Health Policy, London School of Economics and Political Science, London, UK; 3 The Centre for Rapid Evidence Synthesis, College of Health Sciences, Makerere University, Kampala, Uganda; 4 Department of Family Medicine and Public Health, Faculty of Medicine, University of Botswana, Gaborone, Botswana; 5 RSD Institute (Recherche-Santé-Développement), Yaoundé, Cameroon; 6 National Health Research Authority, Lusaka, Zambia; 7 University of Zambia, Lusaka, Zambia

**Keywords:** Health sciences, Research, Science, technology and innovation, Governance, Africa

## Abstract

**Background:**

Health research governance is an essential function of national health research systems. Yet many African countries have not developed strong health research governance structures and processes. This paper presents a comparative analysis of national health research governance in Botswana, Kenya, Uganda and Zambia, where health sciences research production is well established relative to some others in the region and continues to grow. The paper aims to examine progress made and challenges faced in strengthening health research governance in these countries.

**Methods:**

We collected data through document review and key informant interviews with a total of 80 participants including decision-makers, researchers and funders across stakeholder institutions in the four countries. Data on health research governance were thematically coded for policies, legislation, regulation and institutions and analysed comparatively across the four national health research systems.

**Results:**

All countries were found to be moving from using a research governance framework set by national science, technology and innovation policies to one that is more anchored in health research structures and policies within the health sectors. Kenya and Zambia have adopted health research legislation and policies, while Botswana and Uganda are in the process of developing the same. National-level health research coordination and regulation is hampered by inadequate financial and human resource capacities, which present challenges for building strong health research governance institutions.

**Conclusion:**

Building health research governance as a key pillar of national health research systems involves developing stronger governance institutions, strengthening health research legislation, increasing financing for governance processes and improving human resource capacity in health research governance and management.

**Supplementary Information:**

The online version contains supplementary material available at 10.1186/s12961-021-00781-3.

## Background

Research governance has been described as an overarching responsibility of government to ensure effective oversight, coalition-building, system design, accountability and regulation of research for health in both the public and private sectors [1]. The WHO Regional Office for Africa (WHO AFRO) identifies research governance as a core function of a national health research system (NHRS), alongside developing and sustaining resources, financing, and producing and using research [1, 2]. The *World health report 2013* emphasizes that every country needs to have an effective NHRS to set research priorities, develop research capacity, define norms and standards for research, and translate evidence into practice [3]. In Africa, strengthening NHRS is particularly essential to generate research that would inform interventions as well as the new tools to tackle emerging and re-emerging diseases [4].

Past initiatives to improve NHRS in African countries have generally focused on strengthening governance mechanisms, capacity-building and funding [5–8]. These are frequently short-term initiatives, lacking sustained engagement and support. Studies have shown that challenges in health research governance for African countries include inadequate funding for governance activities, misalignment of research funding with national research priorities, lack of optimal coordination among stakeholders and limited health research capacity [6, 9, 10].

Strengthening national health research governance is a long-term investment that requires political commitment and sustainable action, which includes public institutions and policies to regulate and coordinate research institutions and research conduct. Robust and high-performing NHRS have typically integrated governance elements such as national research policies and regulations, institutional structures and systems to guide and support researchers, and funding mechanisms [11]. However, many low- and middle-income countries have historically faced challenges in the development of research governance and management of these elements [9]. This has been attributed to several factors, such as models of research capacity-building that focus primarily on developing individual skills without the necessary national and institutional structures and systems to support the trained individuals, or inadequate funding for research systems strengthening [12, 13].

Several frameworks have been developed to support the design and evaluation of health research governance for NHRS, for research institutions, or for health research funding institutions. The frameworks of Pang et al. [14], WHO’s AFRO NHRS barometer [1] and the Council on Health Research for Development (COHRED) [15] are oriented around needs at the national systems level, to describe and guide the governance of NHRS. Smits and Champagne [16], on the other hand, provide a framework adapted for the governance needs of health research funding institutions. Unlike the other three frameworks, which address activities and roles for health research governance, the WHO AFRO NHRS barometer [1] provides a list of the types of policies and structures that should be in place for health research governance. Table 1 summarizes key features of these frameworks related to governance functions.

**Table 1:** Key concepts from health research governance frameworks

Framework	Scope	Functions	Core elements of health research governance
COHRED Guide to developing and managing effective health research systems [15]	National health research systems and health research institutions	- Governance and management	Policies, priorities and management - Conducive environment and leadership - Ability to optimize resources and international integration - Formalized partnership arrangements - Ethical regulation - Coordination
Smits and Champagne Framework on governance of health research [16]	Health research funding institutions	- Governance	- Accountability and performance - Strategy formulation - Resourcing and instrumentation - Intelligence acquisition - Relationship management
Pang et al. Conceptual Framework for health research systems [14]	National health research systems	- Stewardship	- Define and articulate of a vision for a NHRS - Identify appropriate health research priorities and coordinate adherence to them - Set and monitor ethical standards for health research and research partnerships - Monitor and evaluate the NHRS
WHO AFRO NHRS barometer [1]	National health research systems	- Indicators of governance	- National health policy - National strategic health plan - National health research policy and strategic health research plan - Health research law - National health research management forum - National health research priority agenda - Scientific review committee - National health ethics review committee - Hospital ethics review committees

Looking across these frameworks, some common governance functions can be identified, including policies and legislation, strategic vision and planning, prioritization, coordination, management, and ethical regulation. The Smits and Champagne framework was the only one to include accountability and resourcing within governance functions, perhaps related to its intended use by research funding agencies. The Pang et al. framework is the only one to explicitly include monitoring and evaluation of the NHRS as governance functions, although this is implicit in the COHRED and Smits and Champagne frameworks embedded in management and system optimization.

According to the latest review of NHRS in sub-Saharan Africa using the WHO AFRO NHRS barometer published in 2019, African countries have made some progress on health research governance indicators in the 5 years since the previous assessment [2]. More recently, results from a survey of WHO AFRO Member States specifically reported on which countries have the health research governance structures in place that are recommended by the WHO AFRO regional strategy on research for health with data from 35/47 countries [17, 18]. Neither of these studies, however, provides analysis of how governance arrangements function to support NHRS at a national level.

Indeed, despite the existence of these frameworks and WHO’s NHRS barometer, there have been limited studies of national health research governance in African countries. Sombié et al. present one example of this, however, providing insights on how the West African Health Organization’s (WAHO) collaboration with COHRED helped to strengthen health research governance and management mechanisms in four West African countries [8]. While health research governance is generally the ministry of health’s responsibility in three of the four countries involved in that study, there was limited capacity for this within the health sector. None of the countries had research coordination mechanisms. Sombié et al. [8] found that improvements in areas of governance (e.g. policy development, ethics committees and training, stakeholder mapping, and health research monitoring systems) were made thanks to the participatory design of the project, with each country working on their own priorities defined by them and the shared learning across the implementing teams. The leadership role of a regional organization like WAHO with strong presence and networks in the countries was also a supporting factor for collaboration on health research governance in the countries, although changes in other aspects of NHRS—such as funding, human resources and research use—were highlighted as needing more long-term engagement and context-specific action from local leadership.

Other work has been done at the organizational level, such as studies that look at research governance in research institutions and universities [12, 19, 20]. These studies have examined governance elements such as the presence of institutional research policies, strengthening research management offices and the development of ethics review systems as important functions of governance, or they have focused on specific research areas, such as governance of clinical trials and mental health research [21]. The studies have found some improvements in strengthening of ethics regulatory systems in the African countries, for example through collaborations with support from international partners [19].

Given the fairly limited literature exploring national governance for health research systems, this paper aims to analyse and compare health research governance functions in a set of four African countries—Botswana, Kenya, Uganda and Zambia. It further explores what has supported or challenged the strengthening of health research governance, and stakeholder perspectives on why key functions are important for the governance of NHRS in these countries.

## Country background

We selected the four cases from a larger research project examining health sciences research (HSciR) capacity across the African continent [22]. These four countries are all anglophone former British colonies, which would indicate some potential shared institutional structures and governance. It has been noted that many research institutions were established by the colonial authorities to provide information needed for further exploration and settlement [23]. Yet the countries also have unique features in relation to their history of research and post-colonial experiences. For example, Uganda was home to one of the highest regarded African universities at independence, with Makerere University called “Africa’s Harvard” by some [24]—but post-independence civil conflict and authoritarian regimes dramatically impacted the sector before it could recover from the late 1980s onwards [25]. Kenya on the other hand developed its medical school later than Uganda [23], but established a number of new research institutes over time, including the Kenya Medical Research Institute (KEMRI) in 1979 which has grown to be a globally regarded research centre [26].

Acknowledging the different histories of research institutions in these countries, the current institutional landscape of research institutions is also relevant to the exploration of governance functions, because they are the settings for the training and conduct of HSciR. Table 2 lists the main public and private research institutions, universities and centres of excellence for health research in each country that we have inventoried through document analysis and from discussions with key stakeholders. Whilst this was not a product of a systematic exercise and may not be comprehensive, the list resembles the research institution landscape (for health) inventoried in grey literature (see https://www.commonwealthofnations.org /-Sectors->Country->Education->Research Institutes). Although there is variation in the numbers and types of research institutions, public universities conduct health research in all four countries, and in some instances the universities have also established autonomous research institutes through international collaborations. Specialized research institutes and centres of excellence, particularly in areas of infectious diseases, also constitute important knowledge producers in the institutional landscape.

**Table 2:** Main health research institutions, universities and centres of excellence

Botswana	Botswana International University of Science and Technology The Botswana Harvard AIDS Institute Botswana-UPenn Partnership (BUP) Botswana Vaccine Institute National centre of excellence on infectious diseases University of Botswana WHO Collaborating Centre for Nursing and Midwifery Development
Kenya	Academic Model Providing Access to Healthcare (AMPATH) Alupe Leprosy and Other Skin Diseases Research Centre The Centre for HIV and AIDS Prevention and Research (CHIVPR) East African Kidney Institute (EAKI) Institute of Tropical and Infectious Diseases (UNITID) Kenya Institute for Public Policy Research and Analysis (KIPPRA) Kenya Medical Research Institute (KEMRI) Trypanosomiasis Research Centre (KARI-TRC) University of Nairobi, College of Health Sciences
Uganda	Clarke International University Gulu University Infectious Diseases Institute (IDI) Kampala International University Natural Chemotherapeutics Research Laboratory Makerere University Makerere University Walter Reed Project (MUWRP) Mbarara University of Science and Technology Nkozi University Uganda Industrial Research Institute Uganda Virus Research Institute
Zambia	Centre for Infectious Disease Research Macha Malaria Research Institute Tropical Diseases Research Centre University of Zambia

Although some states are members of the same regional blocks and research hubs, they perform differently according to standardized metrics of national research performance and capacity. That said, they all have reasonably well-established health research activities and infrastructures compared to some other countries in the region. Acknowledging that Kenya and Uganda have a much larger population than Botswana and Zambia, there are similarities and differences in generally accepted per capita indicators of HSciR performance (see Table 3). These indicators were collected within our larger project that searched for comparable data on HSciR capacity and performance across Africa [see Wenham et al. [27] (In Press) for greater detail on these indicators and Mijumbi et al. [28] for a critical reflection on their use and limitations].

**Table 3:** Selected indicators of HSciR performance

Country	GDP (millions, current US$, 2016)^a^	Population (thousands, 2016)^a^	GDP per capita (current US$, 2016)^a^	No. of publications per 1 million inhabitants^b^	No. of first author publications per 1 million inhabitants^b^	No. of trials per 1 million inhabitants^c^	GERD as a % of GDP ^d^	GERD per capita (in current PPP$)^d^	Total R&D personnel per million inhabitants (full-time equivalent [FTE])^d^
Botswana	15,581	2250	6924	784.80	335.96	41.33	0.53728	86.56169	570
Kenya	70,529	48,462	1455	294.79	125.27	13.19	0.78578	19.06104	1029
Uganda	24,079	41,488	580	198.85	84.27	15.69	0.17043	2.93947	42
Zambia	21,064	16,591	1270	166.23	51.89	15.79	0.27819	7.7016	163

^a^ World Bank. Data from 2016 ^b^ Scopus (data publications published between 2008 and 2018) and SciVal (data from 2013 to 2017) ^c^ WHO International Clinical Trials Registry Platform and United States National Institutes of Health clinical trials database. Data from 2018 ^d^ United Nations Educational, Scientific and Cultural Organization Institute for Statistics (category: science, technology and innovation). Data from most recent available year between 2005 and 2016

*GERD* gross expenditure on research and development

Botswana and Kenyan authors are named in more publications (where any author comes from the country) and first-authored publications on health per million inhabitants, relative to those of Uganda and Zambia (with first-author position seen as one potential proxy for research leadership). Botswana and Kenya also spend more on research and development (as a percentage of GDP and per capita) in general (gross expenditure on research and development [GERD] data were not specific to health). Botswana also has a notably higher number of clinical trials per million inhabitants than the other countries, supported by the strong history of HIV/AIDS research collaborations since the early days of the epidemic. While these indicators provide a static snapshot of the state inputs and outputs for HSciR in the four countries, they do not capture the dynamic processes at play behind these metrics, or the research governance underpinning knowledge production.

While Kenya and Uganda have similar health research and development portfolios, Zambia and Botswana are considered to have smaller portfolios mainly focused on clinical trials for infectious diseases [29]. As researchers in institutions from all four countries rely on international funding institutions to support health research, we present data in Table 4 from four leading global health funding bodies provided to each country: the European Commission, the Medical Research Council, the National Institutes of Health, and the Wellcome Trust [30].

**Table 4:** Funding awarded to researchers in each country (2008–2017, in 2017 US$)

Country	Wellcome Trust (United Kingdom-based)	United Kingdom Medical Research Council	United States National Institutes of Health	European Commission
Botswana	184,186.00	–	7,356,998.00	–
Kenya	120,959,548.23	762,618.20	23,965,606.00	2,532,293.26
Uganda	17,943,085.72	40,607,133.72	35,999,665.00	17,641.05
Zambia	15,720.00	–	13,073,179.00	–

Research funding institutions’ websites

Figures reported in this table are in 2017 US dollars based on consumer price index adjustments to account for inflation. Cells with no data (–) represent no funding from these organizations, as we excluded funding for research projects in which the principal investigators were based at non-African institutions, even if these projects included collaborators, field sites or locations of research in Africa

As shown, the external research funding in Kenya and Uganda from these agencies is comparatively higher than for Botswana and Zambia.

While these indicators provide a static snapshot of some of the inputs and outputs for HSciR in the four countries, on their own they cannot capture the research governance systems underpinning knowledge production. In this paper we aim to identify and explore some of the key governance elements and functions of NHRS in our case study countries. The paper draws on both document review to map governance elements, and qualitative research to help explain differences in performance and to draw lessons from their experiences in developing governance of NHRS and promoting HSciR.

## Methods

Data were collected in 2019 through a combination of document analysis and semi-structured interviews. We aimed to both identify the governance structures and policies in place in each country, and then to explore what has supported or challenged the strengthening of HSciR capacity in these countries. For each case, we collected data sequentially from documents and then key informants. In terms of documentary analysis, we reviewed the scientific and grey literature, policy documents and websites of HSciR institutions to identify the HSciR actors and to inventory the HSciR policies and governance mechanisms. Documents for each case were identified through searches of scientific databases, Google Scholar and websites of government agencies (i.e., ministries of health, education, development and science; national research authorities; public health institutes) and research institutions. We looked for literature and other documents that would provide details about key policies, institutions, stakeholders and context of HSciR in each country. Documents were analysed by two of the case investigators (the same ones who conducted the fieldwork) to outline the policy and institutional landscape for HSciR and identify key stakeholders—namely, to describe and contextualize health research governance in each case. The contents of the documents were not included in the thematic analysis, but rather served to inform a mapping and brief description of the policy, regulatory and institutional frameworks for health research governance in each case.

Drawing from the documentary analysis, a stakeholder mapping was conducted in cooperation with local collaborators to identify potential informants in key organizations, whom we invited to participate in the study. Additional names were identified through snowball sampling of key informants. Key informants were from government ministries (health, education and/or science), universities, regulatory bodies, national and international funders, public and private research institutions, international organizations and nongovernmental organizations involved in HSciR. Across the four countries, we conducted 80 interviews with key informants from institutions that fund, conduct and/or govern HSciR in the countries (summary in Table 5). An interview guide was developed which included key themes of investment, funding mechanisms, enabling environments, capacity and sustainability of HSciR in African countries (see Additional file 1 for interview guide). Interviews focused on participants’ experience in carrying out research, or in governing or regulating HSciR. We particularly aimed to identify the facilitators and barriers or other factors that influence HSciR work, policies and regulatory practices in place to support such work, and the challenges in improving HSciR governance in their country. Interviewees were provided with information sheets and provided informed consent for interviews and their recording. Research ethics approvals and national research approvals were obtained from relevant ethics review boards of each country, as well as ethical approval from the London School of Economics and Political Science (see details under Declarations/Ethics approval).

**Table 5:** Study participants categorized by their role in the NHRS

Participant category	Botswana	Kenya	Uganda	Zambia	Total
Regulator/decision maker	1	4	6	5	16
Researchers	17	17	7	12	53
Donors	0	4	3	4	11
Total	18	25	16	21	80

All fieldwork for this paper was led and conducted in each country by two of the authors. Interviews were conducted in English^1^ by members of the research team based in Kenya and Uganda. For Kenya and Uganda, interviews were conducted by the team member for whom it was their home country, while field visits were undertaken to conduct interviews in Botswana and Zambia, working closely with local research collaborators (also two of the authors). The research team, including case investigators conducting interviews, consisted of members based in several African countries and the United Kingdom. All had social science and/or public health backgrounds, with previous research experience and training in qualitative research methods.

As noted, this analysis represents one arm of a larger study focusing on strengthening capacity for HSciR in Africa more broadly. Interview data were transcribed and imported into Dedoose software for thematic coding, focusing in particular on those governance elements which appeared to explain HSciR performance, strengthening or barriers in each setting. The data were coded thematically and semantically independently by research team members using a collaboratively developed coding framework based on the key themes of capacity, governance and context, and several other specific themes (e.g. advocacy, alignment, partnership, funding, leadership). The thematic framework was co-produced by the research team through an inductive approach based on key themes arising from key informant interviews from the ensemble of the fieldwork (across all nine cases in the larger project). Collectively, the research team further revised and refined the framework in response to feedback on provisional coding [31] and team moderation on a sample of data to identify differences in understanding and applying the codes and the identification of additional themes emerging from analysis of the first half of the data (through both remote discussions and a 3-day face-to-face analysis meeting). Upon completion of data collection and coding of the full qualitative interview data set, the research team held nine virtual meetings over 4 months to discuss findings and interpretations of data written up by each case investigator as case studies of what supports the development and strengthening of NHRS in Africa. These meetings served to support a critical approach to the replication in analysis for each case, which is recommended to support trustworthiness of claims in multiple-case replication research design [32].

For this paper we analysed the data coded under a governance category of the larger coding framework for the project as a whole (see Additional file 2 for the coding grid). Codes in the governance category included policies, regulation, legislation and institutions. We coded the data structurally to categorize the interview content wherein informants discussed these concepts [31]. To comparatively analyse this data, we looked for what researchers, funders and decision-makers in the four countries saw as key elements of health research governance at the national level according to their perspectives, and we questioned why and how these were seen to be particularly important as governance functions to strengthen the NHRS.

## Results

We first present a mapping of key policies and institutions for health research governance identified in our case study countries—from documents reviewed and including when these were identified by key informants. Table 6 below gives these details in terms of the legislation, policies, institutions with governance mandates, and the systems for ethical governance.

**Table 6:** Health research governance policies and institutions

Element	Botswana	Kenya	Uganda	Zambia
Science, technology and innovation (STI) legislation	None	- 1977 1st STI Act (amended in 1979) - 2013 2nd STI Act	- Uganda National Council for Science and Technology (UNCST) Act 1990	-1997 National Science and Technology Act
National STI policies/plans	- 1998 STI Policy - 2005 STI Plan - 2012 Research, Science, Technology and Innovation (RSTI) Policy	- 2008 STI Policy	- 2009 STI Policy - 2012 STI plan (2012–2018)	- 2009 STI policy (revised in 2006 and 2009)
Health research legislation	None	- Health Act of 2017 (contains legislation pertaining to health research)	- 2011 Uganda National Health Research Organization (UNRO) Act	- 2013 National Health Research Act
National health research policy	None	- 2019 Research for Health Policy		- 2010 National health research policy
National health research strategic plan	None	- National Research for Health Priorities (2019–2023)	- 2012 UNRO Health Research Policy	- 2008 National health research strategic plan (2008–2011) - 1999 National Health Research Agenda
Oversight of national research overall	- Ministry of Infrastructure, Science and Technology (Department of Research, Science and Technology)	- Ministry of Education (Directorate of Research Management and Development) - National Commission for Science, Technology and Innovation (NACOSTI)	- National Council for Science and Technology (UNCST) - Ministry of Science, Technology and Innovation	- Ministry of Science, Technology and Vocational Training - National Science and Technology Council
Oversight of health research	- Ministry of Health and Wellness, Department of Health Research, Policy and Development, Health Research Unit	- Ministry of Health, Research and Innovation Division, Health Research Office - National Health Research Committee	- Uganda National Health Research Organization	- National Health Research Authority, under Ministry of Health
Ethical review systems (note—institutional review board abbreviated IRB)	- Health Research and Development Committee (HRDC) - IRB, University of Botswana - IRB in some hospitals	- 27 IRBs accredited by National Bioethics Committee (under NACOSTI) - Kenya Medical Research Institute Scientific Ethics Review Unit (KEMRI SERU) - IRB, Amref Health Africa - IRB in some hospitals and universities	- 23 IRBs are accredited by the UNCST	- National Health Research Ethics Board - ERES [Excellence in Research Ethics and Science] Converge - IRB, University of Zambia

### Key emergent governance themes

While our interviews asked questions about what supports and facilitates strengthening HSciR and the personal experiences of individuals involved in both research and government working to build research capacity, we identified four key themes that emerged from our analysis which we focus on in these results. All four of these themes are captured in the frameworks summarized in Table 1 above; however, these particular themes were chosen because they emerged from the analysis as the key processes that mattered most to stakeholders for strengthening and reforming governance that would benefit the NHRS as a whole. This section presents insights and challenges according to four themes in health research governance that we found in our interview data which are part of those commonly identified core elements in the reviews of conceptual frameworks above. They included legislation, coordination, regulation and prioritization.

#### Prioritization of HSciR

A first point that emerged from the analysis is the importance of ensuring that health research aligns with local needs. Participants in all the countries raised a general concern that their governments had not done enough in shaping national health research priorities and consulting in-country stakeholders. Many informants mentioned that the HSciR agenda is often imported from international organizations or from foreign research and funding institutions. This may distort research focus on external priorities when local needs may be different, as explained by a researcher from Kenya.

*The global health agenda is decided at high level, like WHO assemblies. Most researchers tend to respond to the recommendations at that level, which […] poses a challenge because locally we may have priorities that don’t fit into the global and are sometimes ignored*. K17

Each country has attempted to address this in different ways with different structures. In Kenya and Uganda, institutions in the science, technology and innovation (STI) sector have led health research prioritization processes. While the health sector institutions in both countries also conduct similar processes in parallel, there has been little coordination and integration of health research prioritization efforts between the two sectors. In Zambia there is a much longer history of health research priority-setting by health sector institutions than in the other three countries, with the first national health research agenda produced in 1999.

Stakeholder engagement is also a key challenge for priority-setting highlighted by interviewees across the countries. This is important both to define the most relevant health research priorities for the country and to foster endorsement and understanding among stakeholder of the priorities for their use. While there were examples of engagement in a number of these exercises in Zambia, we were not able to identify any NHRS within the countries which appeared to have solved this problem. In Botswana, it also remains a point of frustration, as expressed by one researcher:

*In my opinion, there wasn't much consultation in developing the [health research] agenda. If we had more consultation to develop that, then maybe we [could] say, "Yes, this is what the country needs." But I think it was sort of one-sided thing*. B10

Including stakeholders in establishing national health research priorities was seen as important for capturing local concerns. However, it was also seen as important for facilitating the research itself, by ensuring research undertaken in local areas was accepted as important by the communities. For instance, the strategic research areas in Zambia have facilitated researchers’ entry to communities in regions which are difficult to access for data collection because of the government’s interest in priority questions such as understanding a high prevalence of tuberculosis in the regions.

We did not find any NHRS among these countries with arrangements for prioritization that stood out as exemplary which could offer lessons to others. Yet, one insight which emerged is that integrating responsibility for translating prioritization processes into institutional mandates appears a promising means to begin formalizing expectations and accountability around health research priorities. Informants highlighted those institutional mandates—including the Uganda National Health Research Organization, the National Health Research Authority in Zambia, and the National Health Research Committee in Kenya—as being critical to advancing this function of health research governance.

#### Regulation of HSciR

A second element that informants highlighted was the importance of regulation—in particular of the ethics—of health research. One of the main concerns noted for the ethical regulation of health research is whether there is a clear definition of the institutional mandate for this, and the extent of authority of regulatory bodies. The ethics review systems for HSciR vary across the countries (see Table 6). Without a clearly mandated body for the national oversight of ethical regulation, the fragmentation of regulatory systems for research ethics can be problematic for a NHRS, according to participants. Zambia was the only NHRS with a separate health sector statutory institution, the National Health Research Ethics Board (NHREB), that is centrally mandated for national oversight of ethics policies and guidelines. This differs from Uganda and Kenya, where the responsibility for regulatory oversight is carried out through STI regulatory institutions which accredit the institutional review boards (IRBs) in universities, research institutions or hospitals. In the case of Kenya, the National Bioethics Committee is also responsible for dispute resolution, monitoring and evaluation. Botswana differs from the other cases in that ethical review of all health research is regulated by a committee under the auspices of the Health Research Unit at the Ministry of Health and Wellness.

The differing arrangements for ethics review and oversight could create a number of challenges for coordination. For example, although all of these countries conducted ethical reviews, none of our interviewees identified a centralized repository for monitoring all health-related studies being carried out. Kenya, Uganda and Zambia have specific institutions mandated to review and approve of clinical trials specifically. However, informants noted that a registration system for all HSciR studies (including non-trial research) would be a useful resource for stakeholders and the public, and it could further improve oversight of health research by tracking research project status (i.e. ethics decisions, results, dissemination) over time.

Interviewees stressed the importance of legislation to strengthen ethical regulation. Zambia and Kenya have laws that provide standardized ethical codes for the conduct of health research, including clinical trials [33]. In countries without such legal protections, however, serious concerns could be raised. One decision-maker from Uganda noted a number of “black holes” for which there are no legal frameworks for ethical regulation, such as biobanking and biological material transfer of samples from the community. Uganda has the longest-standing regulatory institution among the four countries, the Uganda National Council for Science and Technology (UNCST), which is actively advising government on policy and managing intellectual property for innovation with other departments, but the gaps in regulations to provide authority for institutions to regulate specific issues was highlighted by a researcher as follows:

*Those regulations help to facilitate or to make the environment research friendly. Some of them do not exist, and yet they should be in place. As a country, we need to look at that area very carefully, what are the necessary research regulations?* U5

Informants in Botswana relayed similar concerns, where despite its long history of HIV clinical trials, there appeared to be no legal frameworks for ethical regulation of research involving human participants or to protect intellectual property.

One of the central challenges that informants shared regarding regulation was the capacity issues for ethical review within IRBs. Participants from Zambia and Botswana reported slow ethics processes due to inadequate reviewer capacity and limited resources to support IRBs. Capacity for ethical review requires specialized experts with knowledge of health research across disciplines, and compensation for time or buyouts for the participation of IRB members. One Zambian researcher (Z04) commented that things were improving in this area with the NHREB but that “we still have a long way to go”. Informants relayed that training of committee members in scientific and ethical review and monitoring of studies with a variety of research designs and methods (including, but not limited to, clinical trials) to improve this function of health research governance is currently missing.

#### Coordination of HSciR

Coordination emerged as a vital third element for health research governance, but informants raised it as a somewhat neglected aspect of health research governance, as most reform efforts have focused on regulation. Ministries of health are often the authorities with the remit for coordination of HSciR, and our findings indicated that coordination of health research can be most effective when there are designated statutory institutions with authority for coordination within legislation.

The situation in Zambia stands out, in that the National Health Research Authority (NHRA) operates as an independent body in the health sector that is responsible for both health research regulation and coordination. Local informants explained that this arose as a result of a long-term process of advocacy—first by research leaders, in collaboration with international partners, encouraging the Ministry of Health to develop legislation on health research that would include coordination as a priority function of governance. This was followed by further advocacy of senior civil servants and policy-makers in the Ministry of Health to other ministries across sectors (including finance) to obtain sufficient budget to implement the statutory bodies. While it has been a long road for the NHRA to come to fruition since the process began in 1997, one Zambian researcher expressed how important this has been:

*The regulatory environment has improved tremendously in 20 years, from nothing to a very rigid system, to a system now that is trying to facilitate and encourage research*. Z07

Although informants highlighted that dedicated statutory bodies for health research governance should ensure that mandates include both regulation and coordination, these do not necessarily need to be carried out by the same institution. For instance, the Zambian experience contrasts with that of Kenya and Uganda, where the national STI bodies in the education sector are primarily responsible for the regulation of HSciR (as discussed in the previous section), while coordination of HSciR has been mandated by law to statutory bodies in the health sector. However, some informants mentioned that the ambiguity of roles and responsibilities between the STI institutions and the health research institutions sometimes leads to duplication of efforts or gaps in leadership. So, for instance, in Kenya, the National Health Research Committee was only established in 2019 to coordinate health research with stakeholders, develop research priorities and advise the government on HSciR policies. A lack of intersectoral collaboration to regulate and coordinate health research was thus a concern of many informants for those countries where governance arrangements were moving from regulation provided through STI institutions (under the auspices of ministries of education or science and technology), towards the inclusion of coordination by the ministries of health or other health sector institutions (see Table 6).

Many informants suggested that coordination of health research by the ministry of health is less than ideal for NHRS because a ministry’s core mission is policy-making. There was a general sense across the countries that ministries of health often lack capacity to coordinate research stakeholders within the evaluation and planning units, where this function generally resides. Rather, statutory bodies, located at arm’s length from the political arena with specialist research expertise, were seen by informants as being more fit for purpose. Thus, coordination was felt to be less effective when it was an additional or secondary responsibility of an agency, as this necessitates dedicated and trained staff without competing with other internal organizational priorities. Coordination was reported as one area of governance that Botswana has struggled to develop and institutionalize within the Ministry of Health and Wellness, primarily due to a lack of dedicated capacity for this role. Thus, while countries might officially mandate coordination to key institutions, performance may be limited without capacity reflected in budgets and human resources.

### Legislation for HSciR

Health research legislation is the final area of governance that emerged from the interviews as important. Multiple respondents highlighted legislation as the point where there could be an opportunity to comprehensively improve many of the elements of governance mentioned in preceding sections. Informants underlined two key potential benefits of legislation in particular—first, how it can formalize institutions and regulations, and second, how it can improve their harmonization.

The first key aspect is the formalization of regulation in a legal framework. Indeed, many informants viewed formal legislation as the gold standard of regulatory practice. We found that many health research governance functions in the included countries did indeed operate on the basis of norms and guidelines. Multiple informants also expressed the view that legal frameworks provide clarity on authority and accountability mechanisms, and that they can offer some protection from political threats to the regulatory environment. Formalization of regulation in law can also bring high-level support for institutionalizing governance and national ownership of the NHRS through parliamentary oversight, secured resources and defined legal status of HSciR stakeholders, practices and issues that can be helpful for raising awareness.

The second key aspect is the harmonization of various regulations within law. Informants from countries with health research laws noted that legislation can also serve the purpose of clarifying the connections and relationships between the legacy of the STI regulatory institutions and the more recent health research statutory bodies (see Table 6). For example, the law in Kenya helped to spell out the links between institutions and stakeholders in the regulatory environment and streamline the regulatory process for researchers, according to one decision-maker.

*The Health Act tries to bring all these regulations dealing with research together, clinical trials being one. [T]here are many different laws and regulations that a researcher has to adhere to when they are carrying out research, and … one of the key complaints they have is that they don’t know [where] they have to go [for what]… NACOSTI [National Commission for Science, Technology and Innovation], ethics committees…* K21

Thus, the experiences of countries like Zambia and Kenya have shown that health research legislation is useful for both constructing dedicated institutions and consolidating the rules in regulatory frameworks. But informants also raised the fact that ensuring this coherence so that the law covers the key issues for health research governance that are at stake in the countries (e.g. ethics, intellectual property, data sharing, biobanking), as well as providing the mandate to institutions for health research financing, coordination and regulation, is a considerable challenge for the process of developing law.

We found that laws in Zambia and Kenya were most emblematic of these two key aspects and had the most comprehensive formal health research legislation of the four countries, while the draft of Botswana’s Health Research Bill (as of 2020) in Parliament reflects a similar comprehensive approach. Zambia appears to have achieved the earliest success of all four countries, being the first to have a national health research policy in 2010 and a Health Research Act in 2013. This process began early with the formation of the National Health Research Advisory Committee in 1997, which gained authority as the proponent of health research legislation. Their success was progressively achieved through sustained advocacy and support from partners. The Zambian experience is unique in our case study countries in terms of having strategically used the support of international collaboration for this.

Advocacy, however, is critical to improving regulatory environments for NHRS in part due to the need for resource allocation. One Ugandan respondent explained that while funders often gave money for research, they rarely did for regulatory development. The trajectory of Zambia shows how incremental change through advocacy for a health research agenda, strategy, policy and health research governance institutions contributed to achieving the adoption and implementation of a comprehensive health research law by building support and coalitions over time. Key individuals (research leaders and policy-makers occupying senior roles in government and research institutions) also facilitated this at critical moments, shaping the policy context to build an enabling environment for HSciR through legislation.

## Discussion

In this study the emerging governance elements from participants’ views and country documents reflect the elements identified in the existing frameworks in the literature regarding health research governance. While the emerging governance elements are similar in all the countries, the countries also have different approaches to and experiences with the governance of health research given their individual contexts. These countries may have some similar historical contexts (e.g. inheriting institutions from a British colonial experience), yet they have differences with regard to political, economic, legal, educational and healthcare systems which are all part of the larger context for the governance of health research. The findings in particular highlight where countries have made progress, such as research legislation and policy development, along with areas like prioritization and coordination that remain areas needing ongoing reflection.

Through the findings we see that legislation in particular stands out as a standard to which countries aspire to formalize their regulatory frameworks and health governance arrangements, and which may help to raise awareness and status of HSciR issues within the broader regulatory environment. However, while health research legislation is seen as a benchmark for governance of NHRS, the process to develop and implement legislation is long and resource-intensive, as examples from our data show that this generally takes a decade or more to achieve. In addition, legislation is reinforced through health research policies and strategic plans. While this is the case for most countries, we observed that countries like Zambia developed health research policy before putting legislation in place. The countries are also at various stages of developing research polices and plans. Another finding is that in some countries like Zambia and Uganda, the health research policies are outdated and there is a slow process in developing new ones. This illustrates that the existing research institutions and their partners continue to do research without guidance from national policies and priorities, which may negatively affect the alignment of research with the population health and health system needs.

One of the main similarities seen across our countries is the intersectoral nature of mandated authority in HSciR governance—although the division of responsibility for HSciR governance between the education and health sector has evolved differently over time in each of the countries. We see the health sectors moving from being strongly regulated by the education sector to self-regulation within either stand-alone health research authorities, as in the case of Zambia, to health research coordination units within the sector that are also responsible for regulation of research in the countries. From the findings, there were strong impressions shared by informants in multiple countries that research units within ministries of health generally do not have sufficient capacity for national oversight and coordination of HSciR, however, and such a role may be better suited to a statutory institution with technical and administrative expertise for this function.

There is significant effort to strengthen research ethics regulation in the countries, with the presence of ethics regulatory structures and guidelines for ethics review in all four countries. The findings show that countries differ in their arrangements for health research ethics and IRB oversight at the national level, whether as a statutory body for health research ethics (Zambia), under the STI regulatory institution (Uganda and Kenya), or within the ministry of health (Botswana). Likewise, other sub-Saharan African countries including South Africa and Nigeria have established national ethics committees to guide the establishment of IRBs [34]. The main challenge is that IRBs are under-resourced, and the institutional capacity for coordination of these regulatory bodies and research institutions is a persisting challenge across the countries.

The findings point to the gaps in research prioritization including slow prioritization processes and lack of stakeholder engagement in priority-setting processes as a fundamental concern for how priorities are developed, defined and used. Frequently, we see prioritization as an exercise that happens on paper, without government or donor funding aligned with national health research priorities. In addition, when researchers use donor priorities to guide their work, they may risk failure to align their studies with the population health and health system needs. COHRED has argued that countries cannot steer research expenditure, promote science and innovation for health, strengthen health research capacity, support research institutions in the use of the priorities, or negotiate with partners for funding without clear national health research priorities [11, 35]. We found that there have been various initiatives to support health research priority-setting in developing countries such as Zambia and Tanzania and four West African countries [8, 11], but overall reviews of national research priority-setting have demonstrated that these processes requires political will, funding and a monitoring system to track progress [36].

Our findings also show that advocacy efforts by local leaders and international partners can be influential in efforts to strengthen HSciR governance when sustained over time and linked to individual NHRS needs in context. Many African countries have received external support to strengthen their NHRS through improvements in national HSciR governance including policy development, research coordination structures and capacity-building, as seen in Zambia and some West African countries [8, 37]. Our findings confirm the lessons from these initiatives that local/external advocacy and international partnerships can be favourable for strengthening research governance. However, there should be national mechanisms for coordination of these partnerships, without which the efforts of the partners may not be recognized and supported.

## Conclusion

Health research governance is an essential component of a strong heath research system as it plays vital role in the enabling environment for HSciR. This study has highlighted key lessons in strengthening research governance in the covered countries as well as gaps to be addressed in order to have a strong research governance structures and mechanisms in the countries. This study was not designed to evaluate whether the improvements in national health research governance arrangements have directly led to increased research productivity. Furthermore, efforts to improve elements of HSciR governance are relatively recent in comparison to efforts to strengthen HSciR capacity. However, the international calls to improve governance of research is based on an assumption that over time improvements to health research governance can also shape research production and utilization when they are part of a systemic approach to NHRS strengthening.

We have highlighted some of the lessons and examples from countries in improving important elements of health research governance at the national level. Improving legal frameworks for the regulation, coordination and prioritization of health research aligned with resources for effective functioning of the governance systems is integral to achieving effective and sustainable NHRS that remains a goal across the African continent [17]. Establishing and strengthening national health research authorities and other statutory bodies with mandates to coordinate and regulate health research across all research institutions and stakeholders is central to this goal. Moving forward it will be critically important to think proactively about the governance of HSciR to improve research outputs and use.

## Supplementary Information

**Additional file 1.** Semi-structured interview guide.

**Additional file 2.** Interview analysis codebook.

## Data Availability

Study materials and anonymized data are available by contacting Clare Wenham (C.Wenham@lse.ac.uk) in the Department of Health Policy at the London School of Economics and Political Science.

## Abbreviations

AIDS: Acquired immunodeficiency syndrome
COHRED: Council on Health Research for Development
GDP: Gross domestic product
IRB: Institutional review board
HIV: Human immunodeficiency virus
HSciR: Health sciences research
KEMRI: Kenya Medical Research Institute
NACOSTI: National Commission for Science, Technology and Innovation
NHRS: National health research system
NHREB: National Health Research Ethics Board
STI: Science, technology and innovation
UNCST: Uganda National Council for Science and Technology
WHO AFRO: WHO Regional Office for Africa

## Footnotes

1 The information and consent form were translated and available in Setswana for participants in Botswana, and participants were offered the opportunity to have the interview in Setswana, as per national ethical guidance.
