# Supplementary material for: Governance of health research in four eastern and southern African countries
Source: Health Res Policy Syst. 2021 Oct 13;19:132. doi: 10.1186/s12961-021-00781-3 (PMC8513324; doi:10.1186/s12961-021-00781-3)
Supplement: Supplementary file 1 — Additional file 1. Semi-structured interview guide. [file 12961_2021_781_MOESM1_ESM.docx]

Additional file 1: Semi-structured interview guide (English)

**Semi-structured guide for interviews with informants about**

**Health Science Research in Africa**

This project investigates how health sciences research (HSciR) capacity can be improved and increased on the African continent. HSciR refers to basic, clinical, and applied science on human health and well-being and the determinants, prevention, detection, treatment, and management of disease. The objective of the project is to identify what promotes an enabling environment for HSciR to thrive - exploring several key areas such as (but not limited to) the policy environment, funding mobilization, and the regulatory and coordinating systems for HSciR conducted in the public and private sectors. For each of our case studies, we the research team will be speaking with the key “funders, doers and regulators” of research across the continent.

In the semi-structured in-depth interviews for each case, we will ask a set of seven general questions to all informants about their individual and institutional experiences with HSciR system in the respective case country to gain insight, through their own work and perspective, into the issues and capacities involved in developing HSciR whether in terms of policy, funding, or regulation. We will ask more specific questions to each type of actor depending on their function (funder, researcher, regulator) and their sectoral sphere (governmental, non-governmental, public, private for profit, international organisation/agency).

Through the analysis of the data collected from these interviews, we aim to learn lessons about what drives and supports HSciR, where are the gaps, and what are the challenges and barriers, and the strategies being used in different case settings to improve and increase HSciR.

**Guide for interviews with researchers and academics**

(in public or private institutions)

***General questions***

1. Who are you? What do you do? Where do you work?  What is your role?

2. What is your experience (doing, funding, regulating/governing) HSciR in [country]?

3. What have been the main facilitators and barriers to your work?

4. What has supported investment in HSciRor capacity for HSciR in [country]? Why has this/ have these been successful?

5. What are the biggest challenges to establishing a vibrant HSciR environment in [country]? How are you addressing these?

6. Have any external factors had a particular impact on the HSciRenvironment in [country]? If so, how?

7. Who do you think is doing well in HSciR?

***Specific questions***

Why have you chosen to work in HSciRhere (in this country, field of HSciR, institution)?

How do you access funding?

What has working in HSciR here (in this country, field of HSciR, institution) allowed you to achieve?

What can be done to improve / increase HSciR?

What are the long terms plans or concerns regarding sustainability of HSciR?

**Guide for interviews with funders, international donors, and philanthropists**

(in public, public-private, foundations, or private not-for-profit institutions)

***General questions***

1. Who are you? What do you do? Where do you work?  What is your role?

2. What is your experience (doing, funding, regulating/governing) HSciR in [country]?

3. What have been the main facilitators and barriers to your work?

4. What has supported investment in HSciRor capacity for HSciRin [country]? Why has this/ have these been successful?

5. What are the biggest challenges to establishing a vibrant HSciR environment in [country]? How are you addressing these?

6. Have any external factors had a particular impact on the HSciR environment in [country]? If so, how?

7. Who do you think is doing well in HSciR?

***Specific questions***

What mechanisms do you use to fund research /or/ invest in HSciRin [country]?

Who or what do you fund or invest in?

Why do you fund research /or/ invest in HSciR (or why not) in [country]?

What would make you increase /or/ begin funding HSciR in [country] (push/pull mechanisms)?

What challenges do you face?

What has kept you here?

What sustainability plans do you have with government to strengthen HSciR?

Do you support HSciR in other countries?

What makes a country attractive environment to invest in HSciR?

**Guide for interviews with government policy-makers**

(in public institutions)

***General questions***

1. Who are you? What do you do? Where do you work?  What is your role?

2. What is your experience (doing, funding, regulating/governing) HSciR in [country]?

3. What have been the main facilitators and barriers to your work?

4. What has supported investment in HSciR or capacity for HSciR in [country]? Why has this/ have these been successful?

5. What are the biggest challenges to establishing a vibrant HSciR environment in [country]? How are you addressing these?

6. Have any external factors had a particular impact on the HSciR environment in [country]? If so, how?

7. Who do you think is doing well in HSciR?

***Specific questions***

To policy-makers about technical matters (i.e. Ministries of Health and/or Education):

What policies and practices are in place to support HSciR?

What are the funding mechanisms for HSciR?

What challenges have you faced?

What are you doing to make HSciR investment attractive for private or international donors / to researchers?

And what would you hope / or / like to do to improve or increase HSciR in your country?

To policy-makers about financing matters/budgets (i.e. Ministries of Finance, parliamentary (health/research) committees, health permanent secretary):

Do you co-finance HSciR?

- If yes, what funding mechanisms do you use to fund research /or/ invest in HSciR?

Who or what do you fund or invest in?

- If no, what would increase your financing?

How do you decide what to spend /or/ invest in HSciR?

**Guide for interviews with private industry**

(e.g. pharmaceutical companies, private health care organisations)

***General questions***

1. Who are you? What do you do? Where do you work?  What is your role?

2. What is your experience (doing, funding, regulating/governing) HSciRin [country]?

3. What have been the main facilitators and barriers to your work?

4. What has supported investment in HSciR or capacity for HSciRin [country]? Why has this/ have these been successful?

5. What are the biggest challenges to establishing a vibrant HSciR environment in [country]? How are you addressing these?

6. Have any external factors had a particular impact on the HSciRenvironment in [country]? If so, how?

7. Who do you think is doing well in HSciR?

***Specific questions***

Do you invest in HSciR in country x and how much? Why?

What funding mechanisms do you use to fund research /or/ invest in HSciR in [country]?

Who or what do you invest in?

What has kept you here?

What would make you increase your investment?

How does this compare with your experience with other countries?

Do you invest in HSciR elsewhere / regionally?

Do you have a long-term strategy for investing in HSciR? Is it country-specific?
