# Supplementary material for: Governance of health research in four eastern and southern African countries
Source: Health Res Policy Syst. 2021 Oct 13;19:132. doi: 10.1186/s12961-021-00781-3 (PMC8513324; doi:10.1186/s12961-021-00781-3)
Supplement: Supplementary file 2 — Additional file 2. Interview analysis codebook. [file 12961_2021_781_MOESM2_ESM.docx]

Additional file 2: Interview analysis codebook

| **Category -  groups of related concepts** | **Code -  descriptive or conceptual label** | **Definitions and examples of data covered by this code** |
| --- | --- | --- |
|  | **1. Advocacy** | Ability and arguments to communicate with and convince senior policy-makers and politicians for HSR support and HSR use (about relevance/significance of health research and for financing) |
|  | **2. Alignment/harmonisation** | Alignment of health research with the context, Re linking the strategic visions to empirical realities on the ground, linking research to population needs / health priorities, linking research to improving health programmes and health system, need for local data and researchers with programmatic view and understanding of implications of their research. |
|  |  | Alignment of health research with other policies, programmes (health, development, etc.) at local, national, regional and/or international levels |
| ***HSR Capacity*** | **3.1 Capacity - Institutional** | research management systems (including coordination of) |
|  |  | research institutions – national research centres, universities, laboratories, and private research institutions |
|  |  | infrastructure, equipment, labs, technology, and tools |
|  |  | curricula (standardised curriculum to ensure HE institutions are giving HSR due attention) |
|  |  | training (quality, mentorship/supervision, competency-based, curriculum, research methods, mentorship/supervision) |
|  | **3.2 Capacity - Research leadership** | Leadership within research institutions (e.g. universities, etc.) support research priorities and provide vision, leadership, and mentoring to develop and institutionalize a research culture. While research leadership is important to grow capacity or manage existing capacity and resources, it may not be from an institution as a whole, and rather linked to individuals. |
|  | **3.3 Capacity - Human** | Individual technical capacity for grant writing, research methods |
|  |  | human resources: training, retention/attractiveness, ethics, next gen, right mix/missing skills sets, |
|  | **3.3.a Capacity - Human - Motivation** | The reasons why individual researchers remain dedicated and driven to pursue a career in research in their given country/institution. Some examples of these include specific interests, commitment to country/community/research area, passion, sense of purpose, sense of duty to develop health research in country, students, family, etc. |
|  | **3.4 Capacity - other** | culture of scientific research (perception of research and its value, understanding the research process, reflection, publication, strong research community, confidence, institutionalisation of research practices) |
|  |  | interdisciplinary/multidisciplinary research for health |
|  |  | competitive environment / competitive according to international standards |
|  |  | availability (how much capacity is available in the given jurisdiction at any given point in time) |
|  |  | building (efforts to build the capacity to meet the particular country’s needs) |
|  | **4. Collaboration / Partnership / Networking** | (between stakeholders and partners: national / international, NGOs, international agencies, funders, local and international universities, private sector) |
|  |  | skills/competencies transfer |
|  |  | agenda-setting/influence on agenda of health research in Africa |
|  |  | financing |
|  |  | networks/networking - learning, exchange, training, support, mobility |
|  |  | North-South, South-South, Anglophone-Francophone, African region, sub-regional (e.g. Indian Ocean Commission) |
|  |  | transversal approaches – breaking silos (disciplinary, sectoral, but also disease-specific/vertical programmes) |
|  |  | conferences, seminars, or other partner meetings, and stakeholder platforms in the country and internationally |
|  |  | institutional or individual arrangements |

|  | **5. Community participation/engagement** | relevance, understanding, and acceptability of research by community |
| --- | --- | --- |
|  |  | community involvement in problematising, developing, conducting, analysing, translating, or using research |
| ***Context*** | **6.1 Sociocultural** | social status; language; cultural beliefs, values, and traditions |
|  | **6.2 Political** | politics, political change, political crisis |
|  | **6.3 Economic** | economic status, change |
|  | **6.4 Epidemiological** | health/disease status and distribution, emerging and re-emerging diseases (NTDs) |
|  | **6.5 Geographical** | physical environment, roads/transport, climate/weather |
|  | **6.6 Technological** | electricity, phone/internet, also new technologies for research |
|  |  | Additional cost of research related to the context: access to advanced equipment / inputs (purchase, tariffs, transport, maintenance) / field work needs in challenging contexts |
|  | **7. Crises - health** | HIV, Ebola, etc. |
|  |  | outbreaks of measles, pneumonic plague, cholera, etc. |
|  |  | severe undernourishment |
|  |  | climate change and heath |
|  |  | major public health crises |
|  | **8. Funding** | resource mobilization, management and sustainability |
|  |  | financing / funding mechanisms (domestic and international) |
|  |  | e.g. budgets, grants, calls for proposals, scholarships, aid/cooperation instruments, etc. |
| ***HSR Governance*** | **9.1 Governance - Policy** | policies, plans, and other strategic guidance (presence of national and institutional policies) |
|  |  | priority/agenda setting, policy change, policy gaps, policy implementation/evaluation |
|  | **9.2 Governance - Legislation** | laws, decrees |
|  | **9.3 Governance - Regulation** | regulatory capacity |
|  |  | coordination mechanisms |
|  |  | ethical regulation and governance |
|  | **9.4 Governance - Institutions** | structures and institutional change |
|  |  | coordination structures |
|  | **9.5 Governance - other** |  |
|  | **10. Ownership** | The sense of ownership over any aspect of research or the research process or outcomes (agenda, ideas, results, resources, etc.). |
|  | **11. Political will/Leadership** | prioritisation given to health research |
|  | **12. Private sector involvement** | private-for-profit industry sector |
|  | **13. Research use / knowledge translation** | packaging |
|  |  | use / application / adoption |
|  |  | visibility and dissemination (including scientific publications and conferences, multi-stakeholder platforms, and community health workers, district health centres, and the public) |
|  |  | access, availability to research - including coordination of research and knowledge – i.e. database/inventory clearing houses for all research in the country |
|  |  | impact |
|  |  | documentation of KT and examples of research use |
|  | **14.Other** |  |
